# A microwave synthesized mesoporous carbon sponge as an efficient adsorbent for Cr(vi) removal†

**DOI:** 10.1039/c8ra00012c

**Published:** 2018-02-19

**Authors:** Yan-Jun Liu, Shan Liu, Zhi-Wen Li, Ming-Guo Ma, Bo Wang

**Affiliations:** Engineering Research Center of Forestry Biomass Materials and Bioenergy, Beijing Key Laboratory of Lignocellulosic Chemistry, College of Materials Science and Technology, Beijing Forestry University Beijing 100083 PR China mg_ma@bjfu.edu.cn; Key Laboratory of Pulp and Paper Science & Technology of Ministry of Education/Shandong Province, Qilu University of Technology Jinan 250353 PR China

## Abstract

Mesoporous carbon materials have recently attracted immense research interest because of their potential application in water purification fields. Herein, we report the synthesis of a mesoporous carbon sponge (MCS) from a supramolecular microcrystalline cellulose–polymer system triggered by microwave-assisted treatment. Benefiting from the three-dimensional (3D) interconnected mesopores and an evenly distributed ball-like protuberance on the inner surfaces of the macropores, the MCS exhibited a high adsorption capacity (93.96 mg g^−1^) for fast Cr(vi) removal within 5 min. Additionally, the MCS can be regenerated and reused after the adsorption–desorption process, and maintained an adsorption capacity of ∼86% after 10 cycles. The high adsorption capacity, significantly reduced treatment time, and reusability make the MCS promising for the purification of wastewater on a large scale.

## Abstract 2

Mesoporous carbon sponge (MCS) is successfully prepared from a supramolecular microcrystalline cellulose–polymer system triggered by microwave-assisted treatment. The MCS exhibits high adsorption capacity for fast Cr(vi) removal within 5 min.

## Graphical abstract

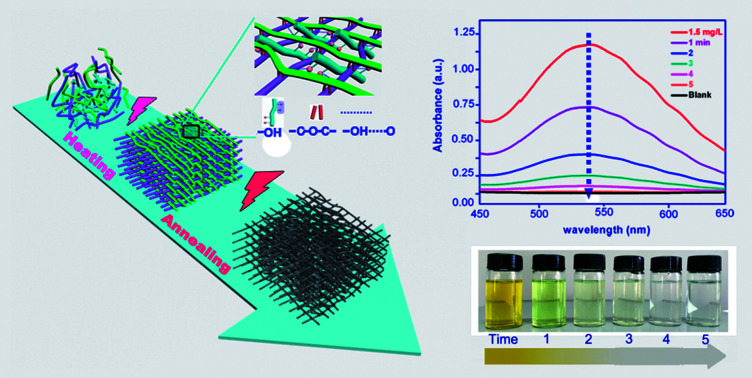

## Introduction

Environmental problems have been receiving more and more attention in China, and include a large amount of wastewater containing heavy metals (Cr(vi), Cd(ii), Hg(ii), and Pb(ii)) being released directly into surface water and groundwater from unregulated industries. Among those heavy metal species, Cr(vi) has been classified as a carcinogen and mutagen due to its acute toxicity to most organisms and strong carcinogenic properties.^1^ Although recent advances in water purification technologies have allowed researchers to remove Cr(vi) from wastewater, such as cyanide treatment,^2,3^ electro-chemical precipitation,^4^ photocatalytic reduction^5–7^ membrane filtration^8,9^ and adsorption,^10–13^ there are still some problems requiring improvement. For example, the cyanide treatment may result in highly toxic intermediates. The electro-chemical precipitation may induce second-pollution, requiring additional treatment processes. Furthermore, the lower adsorption rate of heavy metal ions and narrow photoabsorption region in photocatalytic reduction are not suitable for decontamination at a large scale. Therefore, development of environmentally friendly and efficient methods is of great importance for the Cr(vi) removal from wastewater.

Mesoporous carbon materials with high specific surface area and hierarchically porous networks are alternatively efficient adsorbents for the removal of heavy metals. It has several variants, including activated carbon (AC),^14^ magnetic carbon nanocomposites (MCNs),^15–18^ carbon nanofibers (CNFs),^19^ carbon nanotubes (CNTs),^20,21^ carbon membranes (CMs),^8,9^ carbon sponge (CS),^22–24^ and carbon aerogel (CA).^25–29^ Until now, some successful strategies such as the thermal decomposition,^12^ electrochemical deposition,^30,31^ layer-by-layer technique,^20,32^ self-assembly,^33^ and synergistically assembly^29^ were employed for the synthesis of advanced mesoporous carbon materials. Among them, microwave-assisted thermal treatment could not only selectively couple with the intermediate in a multiphase reaction system but also overcome the high activation energies for product formation.^34^ Moreover, the microwave heating reduces the overall thermal gradients in the reaction and thus yields more uniform products.^35^ Hence, microwave-assisted thermal treatment, as an economic, more efficient, and green method, is an alternative technology for preparing mesoporous carbon materials. However, as far as we know, the preparation of MCS from a supramolecular microcrystalline cellulose–polymer system *via* synergistically assembly triggered by microwave-assisted treatment has not been reported yet.

**Fig. 1:**
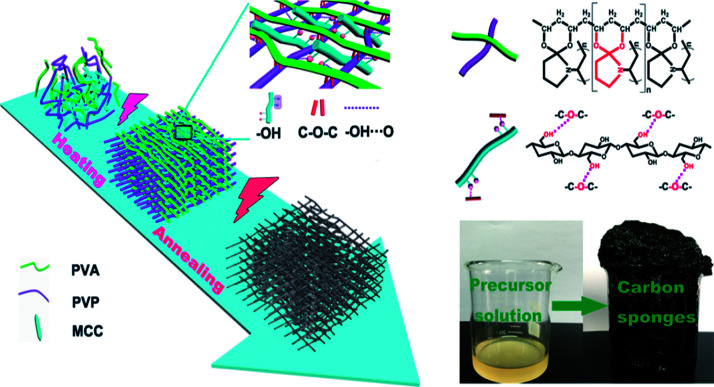
Schematic illustration of the formation of carbon sponge (PPM-C) (inset: photo of the carbon sponge).

In this work, an environmentally friendly strategy was applied to prepare mesoporous carbon sponge (MCS) with three-dimensional (3D) interconnected structures by the microwave-assisted method using microcrystalline cellulose (MCC), poly(vinyl alcohol) (PVA), and poly(vinyl pyrrolidone) (PVP) (Fig. 1). The molecular chains of PVA, PVP, and MCC served as carbon sponge precursors, which undergo synergistically assembly upon the simultaneous covalent polymerization and physical interaction triggered by microwave-assisted heating/annealing treatments. The microwave-assisted synthetic process is simple and time-saving, and thus the product can be easily manufactured in a large-scale. Subsequently, we focused our efforts on studying Cr(vi) removal through the MCS. The as-prepared MCS exhibited exceptionally high adsorption capacity of 93.96 mg g^−1^ and fast Cr(vi) removal from wastewater only within 5 min.

## Experimental

### Materials

All chemicals used here were of analytical grade and used as received without further purification. All experiments were operated under an ambient atmosphere. Microcrystalline cellulose (MCC, molecular weight of 34 843–38 894 degree of polymerization (DP, DP = 215–240)) of a commercial reagent was purchased from Sinopharm Group Chemical Reagent Co., Ltd. Poly(vinyl alcohol) (PVA, ∼88% hydrolyzed, DP = 1750 ± 50) was purchased from Sinopharm Chemical Reagent Co., Ltd. and poly(vinyl pyrrolidone) (PVP, K-30) was purchased from Xilong Chemical. Potassium dichromate (K_2_Cr_2_O_7_), sulfuric acid (98%), sodium hydroxide (NaOH), and sodium chloride (NaCl) were purchased from Beijing Chemical Works.

### Preparation of carbon sponges

For the preparation of the MCS, typically, solution A was prepared by dissolving 10 g PVA in 115 mL distilled water, under vigorous stirring (800 rpm) at 85 °C for 15 min. And solution B was prepared by dissolving 7.5 g PVP in 42.5 mL distilled water under ultrasonic wave (100 W) for 10 min. Two different strategies were used to prepare the MCS: (a) the solution A and B were mixed uniformity of their volume ratio of 7 : 3, and the sulfuric acid (*ω* = 10 wt%, 1.5% of the reaction mixture) was added to the reaction mixture under vigorous stirring, obtaining the precursor solution. The precursor solution was then poured into a glass beaker (100 mL) for immediate exposure to microwave energy at 300 W using UWave-1000 (Shanghai Sineo) in a high purity N_2_ atmosphere for 5 min, and then microwave-assisted annealing at 900 W for 10 min, obtaining the MCS and the resulting product was named PP-C. (b) The other MCS was fabricated by the same strategy as in (a), and the only difference is that the precursor solution consist of solution A and B, the sulfuric acid, and MCC (1.5% of the reaction mixture). The resulting product was named PPM-C (Table S1†).

### Characterization

The morphology of the samples was characterized using scanning electron microscopy (SEM, SU8010) with a field emission gun, operated at an accelerating voltage of 10.0 kV. The energy-dispersive X-ray spectra (EDS) attached to the scanning electron microscopy (ZEISS EVO18) was used to analyze the composition of sample (scanning area: 4 × 5 μm^2^), operated at an accelerating voltage of 15.0 kV (takeoff angle = 35.0° elapsed livetime = 9.2). Fourier transform infrared (FT-IR) spectroscopy was obtained on Thermo Scientific Nicolet iN10 FTIR Microscope (Thermo Nicolet Corporation, Madison, WI, USA), which was equipped with a liquid nitrogen cooled MCT detector. The X-ray photoelectron spectroscopy (XPS) measurements were performed on Thermo Scientific Escalab 250Xi XPS system (Thermo Fisher Scientific Ltd., UK) using Al Kα source. The specific Brunauer–Emmett–Teller (BET) surface areas were measured on a Quantachrome Quadrasorb Station 1 by nitrogen adsorption at 77.3 K and the pore-size distributions was measured on a Quantachrome Quadrasorb Station 1 by Barrett–Joyner–Halenda (BJH) pore size desorption isotherms at 77.3 K (sample weight: 0.1041 g and analysis time: 490.2 min).

### Adsorption experiment

The standard solutions of Cr(vi) with different concentrations (1.5 and 4.0 mg L^−1^) were obtained by dissolving potassium dichromate in distilled water. Cr(vi) solutions were treated with 1.0 g L^−1^ MCS to study the removal capacity. Briefly, the mixture was placed in an incubator shaker (100 rpm) at room temperature for 5 min. Then, the MCS was removed gently with forceps. The clear solutions were collected and subjected to colorimetric analysis to determine the remaining Cr(vi) concentration. The pH study was conducted at different pH values from 2 to 11, the pH value was adjusted by using hydrochloric acid (using 1 mol L^−1^ HCl to adjust pH from pH 2–4 and concentrated HCl for pH = 1) or sodium hydroxide solutions (1 mol L^−1^). For colorimetric analysis, the aforementioned clear solutions (5.25 mL) were taken into test tubes, then *o*-phosphoric acid (0.50 mL, 4.5 M) and DPC (0.25 mL, 5 g L^−1^) were added. After incubation at room temperature for 30 minutes for color development, the absorbance of the samples was measured in a UV-Vis spectrophotometer (Techcomp UV2310II) at *λ*_max_ = 540 nm (Fig. S1a–f†). To evaluate the efficiency of different adsorbents, removal percentage (RP, %) is introduced as a criterion, which can be calculated using eqn (1):1
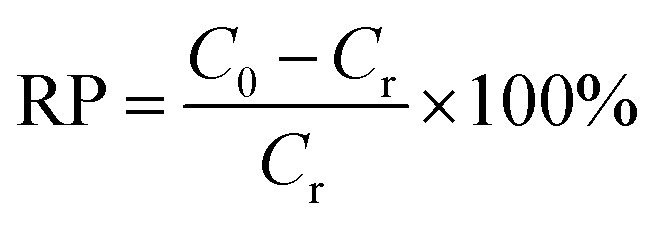
where *C*_0_ is the initial concentration of the Cr(vi) solution and *C*_r_ represents the remaining Cr(vi) concentration after treatment. The adsorption capacity (*q*_e_, *m* of Cr(vi) per g of adsorption) of Cr(vi) was calculated by the following equation eqn (2):2
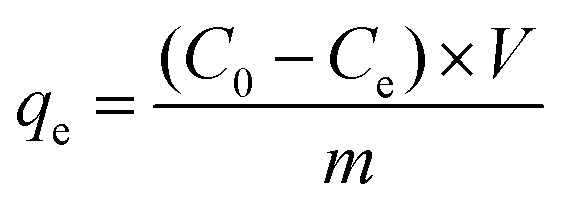
where *C*_0_ (mg L^−1^) and *C*_e_ (mg L^−1^) are the initial and the equilibrium concentrations of dye. *V* (L) is the volume of aqueous solution, and *m* (g) is the weight of the adsorbents.

### Desorption experiment

After adsorption experiment, the MCS was put into a conical flask containing 50 mL NaOH (15 wt%) and 50 mL NaCl (15 wt%). Then, the conical flask were stored in shaking incubator (100 rpm) at 25 °C for 24 h, and then taken out the MCS from the conical flask and dried it to be reused. At last, the waste liquid from the MCS after adsorption treatment was collected and sorted for centralized treatment.

## Results and discussion

### Microstructure investigation of MCS

**Fig. 2:**
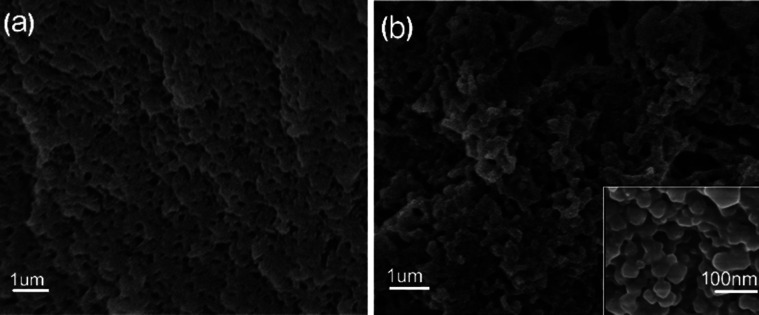
SEM images of (a) PP-C and (b) PPM-C (PP-C and PPM-C represent the composition of the carbon sponge containing PVA/PVP and PVA/PVP/MCC, respectively).

Fig. 2a shown the scanning electron microscope (SEM) microstructure of the PP-C after microwave-assisted heating/annealing treatments. The PP-C exhibited a sponge-like structure with homogeneous pores. The pores arranged sparsely, and the pore diameter was mostly centered at hundreds of nanometers. In contrast, the PPM-C exhibited a sponge-like structure with 3D interconnected pores by microscopic observation (Fig. 2b). Taking a closer view on the inner surfaces of the pores in PPM-C, the inset of Fig. 2b, it is interesting to observe a “shiatsu plates” structure decorated with ball-like protuberance and that is uniform distribution. We attribute the 3D interconnected pores to the microwaves selective heating: the reactants (polymers and MCC) with different dielectric constants led to the asynchronism of microwave power absorption value (Table S2†),^36,37^ thus resulting in different direction of the steam migration in the transition state. Similarly, the 3D interconnected mesopores also derive from the channels remained of the steam migration. Their macro/micro scale structures provide a high surface area, a high surface-to-bulk ratio, and in combination these features are suitable for the removal of heavy metal ions from water.

### Chemical structure of MCS

**Fig. 3:**
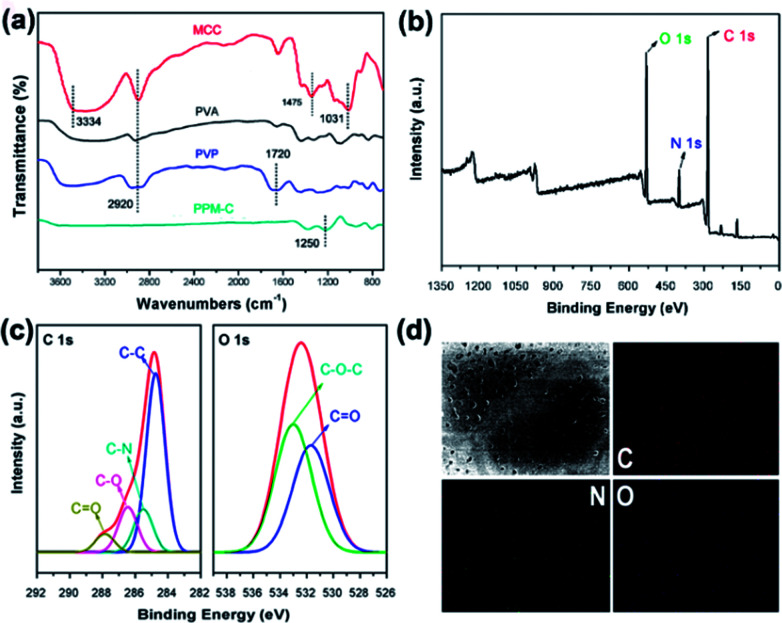
(a) FT-IR spectra of the MCC, PVA, PVP, and PPM-C, (b) the whole survey XPS spectrum of the PPM-C, (c) C 1s and O 1s XPS spectrum of the PPM-C, (d) energy dispersive X-ray images with the corresponding mapping of C, N, and O elements.

The Fourier transform infrared (FT-IR) spectra of MCC, PVA, PVP and as-prepared PPM-C were presented in Fig. 3a. The basic characteristic peaks of MCC appeared at 3334 cm^−1^ (O–H stretching vibration), 2920 cm^−1^ (C–H stretching vibration), 1475 cm^−1^ (C–H framework vibration) and 1031 cm^−1^ (C–O stretching vibration). The basic characteristic peaks of PVP appeared at 1720 cm^−1^ (C=O stretching vibration of the pyrrole ring). Comparing the FT-IR spectra of MCC, PVA, PVP, and PPM-C, the spectrum ranged from 4000 to 1700 cm^−1^ without stretching vibration peaks in PPM-C, which explained that the chains of MCC, PVA and PVP were degraded to carbon skeleton through the microwave-assisted annealing process. The characteristic absorption band around 1350 cm^−1^ could be assigned to the absorption peak of the stretching vibration of ether bond (O–C–O).^38^ The ether bond derives from the ketalization reaction between ketones of PVP (PVP–C=O) and alcoholic hydroxyl groups of PVA (PVA–OH) (Fig. S2†).^39^ And X-ray photoelectron spectroscopy (XPS) measurement was carried out to further investigate functional groups presented on the surface of as-prepared PPM-C. The whole XPS survey spectrum of the as-prepared PPM-C (Fig. 3b) exhibited three obvious peaks at 284.84, 399.33, and 532.3 eV, which were attributed to C 1s, N 1s and O 1s, respectively. The C 1s XPS spectrum (Fig. 3c) can be further deconvoluted into three carbon states at 284.84, 285.40, 286.15 and 288.7 eV, which were attributed to C–C, C–N, C–O and C=O respectively.^40^ Moreover, the peak of double bond (C=O) near 289 eV disappeared, thus further verifying the ketalization reaction between ketones of PVP and alcoholic hydroxyl groups of PVA. The O 1s spectrum (Fig. 3c) showed two peaks at 533.2 and 531.6 eV, which was assigned to O–C–O and C=O bands. The C–O–C peak indicated the successful ketalization reaction between ketones of PVP and alcoholic hydroxyl groups of PVA, which was further confirmed by the energy dispersive X-ray spectra (EDS) images with the corresponding element mappings in Fig. 3d. These results showed that the presence of these key functional groups (C–C, C–N, and C–O) on the surface of the as-prepared PPM-C without being broken,^41^ indicating that the carbon skeleton morphology was well maintained after the annealing process. Namely, the precursors were initially heated by the microwave-assisted heating, in which the PVP chain was linked to PVA chain by ether bonds (O–C–O). Meanwhile, MCC as a filling agent was bound together with the polymer network by hydrogen bonds, obtaining a sol-like solution. Then, the sol-like solution was annealed to obtain carbon sponge (PPM-C) with the ordered carbon skeleton. The schematic formation mechanism of the PPM-C was also illustrated in Fig. 1.

### The interfacial behavior of MCS

**Fig. 4:**
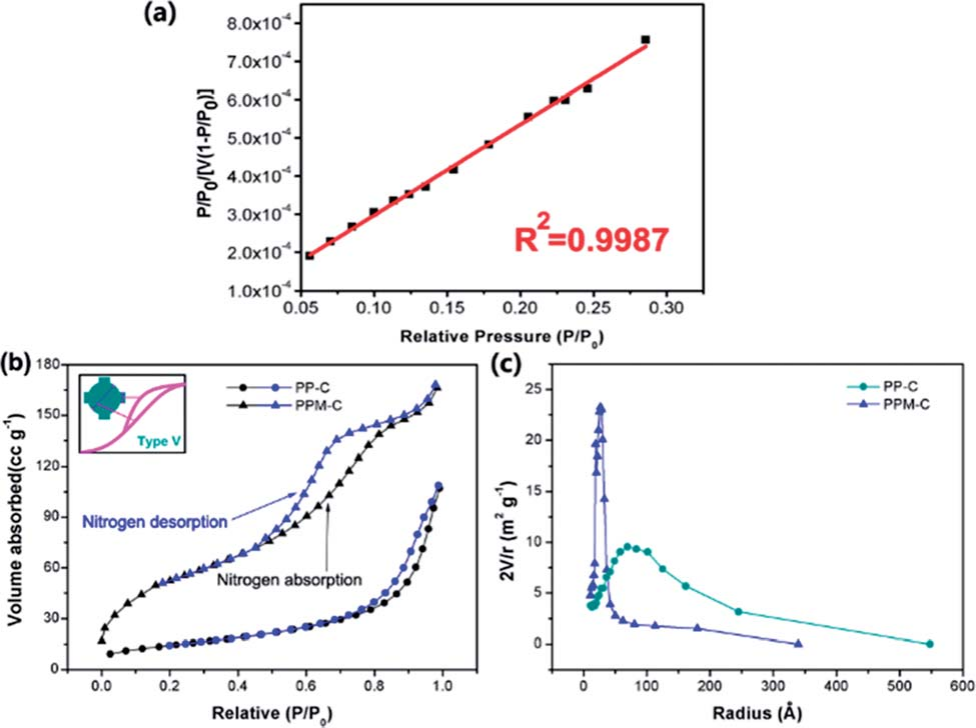
(a) Linear relationship between the relative pressure and adsorption, (b) nitrogen absorption–desorption isotherms curves of PP-C (circle) and PPM-C (up triangle) calculated using the BET method (black: nitrogen absorption curve, blue: nitrogen desorption curve, inset: the type V of gas adsorption isotherms), (c) the pore-size distribution of PP-C (circle) and PPM-C (up triangle) calculated using the BJH method.

Specific surface area and pore size are critically important for mesoporous carbon materials as adsorbents for the removal of heavy metal ions from water. N_2_ physisorption isotherms were applied to evaluate the surface properties of the carbon sponge. In order to determine specific surface area and pore size, a linear standard curve relating has been made (Fig. 4a). As can be seen in Fig. 4b, N_2_ sorption isotherms of the PPM-C are the representative type V reversible sorption (the curve in the inset), indicative of a typical mesoporous network structure.^42^ This is confirmed by the Barrett–Joyner–Halenda (BJH) pore size distribution curves calculated from the desorption branches (Fig. 4c). It is clearly shown that the pore radius size of PPM-C was highly centered at ∼25 Å compared with PP-C at ∼70 Å, and their characteristic diameter of ∼50 Å was in the mesoporous range of 20–500 Å,^41^ verifying the carbon sponge with 3D interconnected mesoporous structures. Moreover, the PPM-C exhibited the high specific surface area of 310.10 m^2^ g^−1^, which is much higher than 99.73 m^2^ g^−1^ for PP-C. One of the major reasons for the increased specific surface area of the PPM-C is that the mismatch between MCC and their surrounding polymer matrix induced the surface elastic instability, which lead to the ball-like protuberances in the process of cooling for increasing the surface area per unit weight. Importantly, the adsorption–desorption curves of PPM-C exhibited discernible steps over the entire relative pressure range (Fig. 4b). This result may be interpreted that the adsorption process is initially similar to that on macroporous solids, but at higher pressures the amount adsorbed rises steeply due to the capillary condensation in mesopores.^43,44^ The amount adsorbed rises steeply of PPM-C from the relative pressure value at 0.6, compared with that of PP-C at 0.9, verifying that the capillary condensation of PPM-C in mesopores is particularly prominent. Moreover, the adsorption isotherms for PPM-C exhibited high-pressure hysteresis loops (with a closed hysteresis loop), indicating the adsorbent without swelling and chemisorption during the adsorption process. Consequently, the 3D interconnected mesopores and an evenly distributed ball-like protuberance on the surface enable the PPM-C to achieve high specific surface area and multilevel structures for the purification of wastewater.

### Cr(vi) removal by MCS

**Fig. 5:**
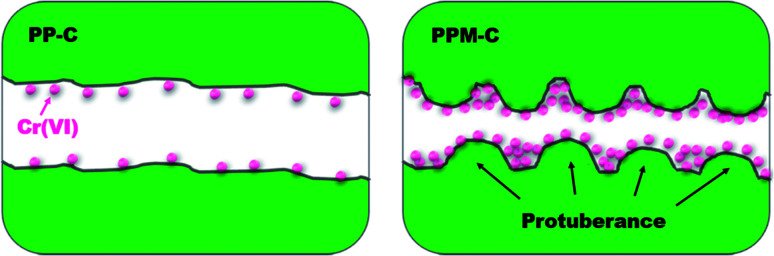
Schematic illustration of carbon sponge for Cr(vi) removal (PP-C with a smooth interface and PPM-C with a rough interface decorated with ball-like protuberance) (cross-sectional view).

The obtained PP-C and PPM-C were used as adsorbents to remove Cr(vi) from pre-prepared Cr(vi) aqueous solution. As depicted in the Fig. 5, the adsorption mechanisms of PP-C and PPM-C for Cr(vi) are different. The Cr(vi) on PP-C with a smooth interface is a single-layer adsorption just like active carbon benefited from high specific surface area. In contrast, the high adsorption capacity of Cr(vi) on PPM-C benefits from the synergistic effect of the increased specific surface area and multilevel structures that consist of 3D interconnected mesopores and a rough interface decorated with ball-like protuberance.

**Fig. 6:**
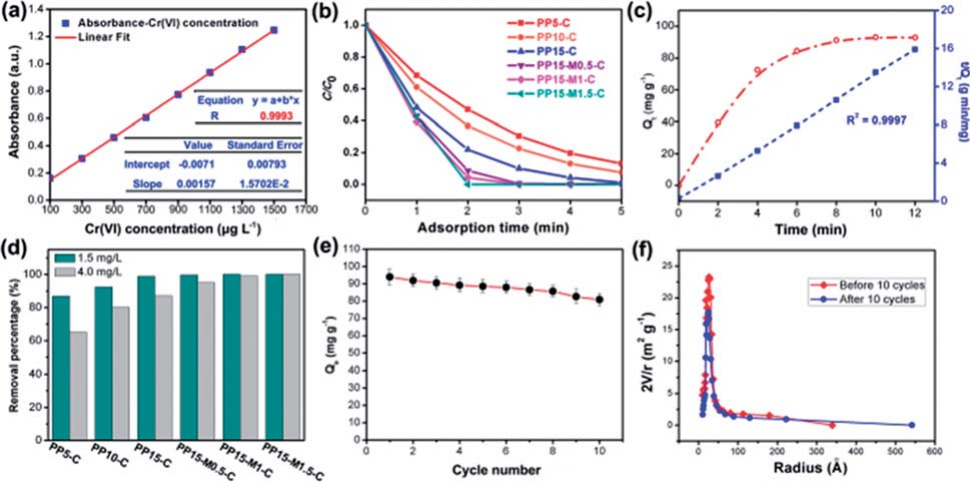
(a) Linear relationship between the Cr(vi) concentration and UV-Vis absorption tested at room temperature. (b) The Cr(vi) removal performance of different carbon sponge (adsorbent: 1.0 g L^−1^, Cr(vi): 1.5 mg L^−1^, where *C*_0_ represents the initial concentration of the Cr(vi) and *C* represents the concentration of the Cr(vi) after adsorption), and (c) kinetic adsorption data plots of Cr(vi) adsorption by PPM-C: Cr(vi) removal rate *q*_*t*_*vs.* time (open circle) and the transformed rate plot *t*/*q*_*t*_*vs.* time (solid square). (d) The RP of different carbon sponge *vs.* time at different initial concentrations of Cr(vi) (adsorbent: 1.0 g L^−1^), (e) adsorption capacities of PP15-M1.5-C as a function of repeated adsorption–desorption cycles for the Cr(vi) removal. (f) The changes of pore-size distribution of the PP15-M1.5-C before and after 10 adsorption–desorption cycles.

In order to determine the change of trace concentrations of Cr(vi), a linear standard curve relating the UV-Vis peak intensity at 540 nm has been made (Fig. 6a). The residual Cr(vi) concentration after treatment can be calculated from the standard curve. Fig. S4a and c† show the UV-Vis absorption of the 1.5 mg L^−1^ and 4.0 mg L^−1^ Cr(vi) solution and the solutions after treatment with PP-C and PPM-C in an incubator shaker for 5 min, which are consistent with the standard curve (Fig. 6a). After treated with different PP-C and PPM-C in an incubator shaker for 5 min, the peak intensity decreased gradually with increasing the concentration of PVP and MCC. Moreover, the peak intensity after treated with carbon sponge with MCC decreased significantly, compared with that without MCC. Upon the subsequent EDS measurement, Fig. S3a–f and g–l† exhibit the composition of PPM-C before and after Cr(vi) removal, respectively. One can observe that the Cr map (Fig. S3l†) of the PPM-C after Cr(vi) removal appeared chromium fluorescent points, verifying PPM-C for Cr(vi) removal from another side. The Cr(vi) removal percentage treated by different PP-C and PPM-C is shown in Fig. 6b. It can be found that the Cr(vi) RP of PPM-C with different contents of MCC are all close to 100% within 5 min while the highest Cr(vi) RP of PP-C barely reach to 92%. With a very short treatment time of 5 min, the removal capacity of PPM-C reaches 93.96 mg g^−1^ by physical adsorption, which is much higher than those of previously reported other materials (Table S2†). Furthermore, the PPM-C exhibits gradually stronger adsorption properties with increasing the MCC content, indicating that MCC as a filling agent is critical for highly efficient Cr(vi) removal. Therefore, PPM-C will be used as the model compound to systematically study the Cr(vi) removal kinetics and mechanisms. The kinetics of the adsorption describing the Cr(vi) uptake rate is one of the important characteristics which control the residence time of adsorbate uptake at the solid–liquid interface. Hence, in the present study, the kinetics of Cr(vi) removal was carried out to understand the adsorption behavior of the as-prepared PPM-C. Fig. 6c shows the Cr(vi) adsorption data over PPM-C at different time intervals (open circle). By evaluate the suitability of different models including pseudo-first-order,^45^ pseudo-second-order,^46^ Elovich^47^ and intraparticle diffusion,^48^ the kinetics of Cr(vi) removal for the pseudo-second-order model was the highest correlation coefficient of *R*^2^ = 0.9997 (Fig. 6c, square symbol curve), indicating that pseudo-second-order model provides an excellent correlation for the adsorption of Cr(vi) on PPM-C.

The pseudo-second-order model equation is given as eqn (3):3
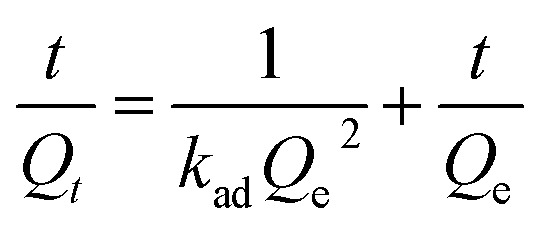
where *Q*_*t*_ is the solid-phase loading of Cr(vi) in the adsorbent at time *t*, *Q*_e_ is the adsorption capacity at equilibrium, and *k*_ad_ is the rate constant of adsorption.

**Fig. 7:**
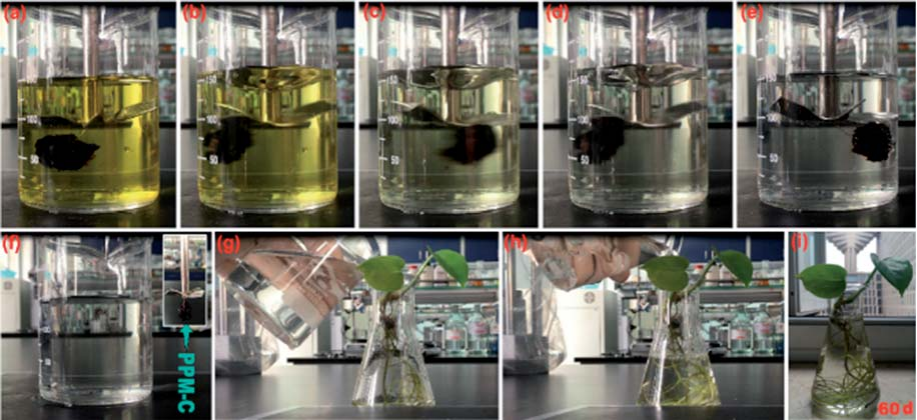
(a)–(e) Color changes of the Cr(vi) solutions during the adsorption process by the PPM-C, and (f) the PPM-C can removed from the water with morphological integrity. (g) and (h) The Cr(vi) solution after treatment by PPM-C be used for water culture experiment, and (i) the plant grown well after 60 days.

Fig. 6d shown that the RP of PPM-C with different components all reached to ∼100% at a low concentration of Cr(vi) (1.5 mg L^−1^), while the RP of PP-C increased from 86.91% to 98.87% upon an increase of concentration of PVP. To further investigate the performance of Cr(vi) removal in a high concentration conditions, PP-C and PPM-C were tested in a Cr(vi) solution of 4.0 mg L^−1^. It is obvious that the RP of PP-C decreased than that of 1.5 mg L^−1^ (65.32–87.54%). By comparison, the RP of PPM-C with a slight decrease further displayed the superior performance of PPM-C for Cr(vi) removal compared with PP-C. To further investigate the recyclability of the MCS, recycled adsorption–desorption experiments were carry out. After 10 cycles, the adsorption capacity of the MCS still maintained ∼86%, indicating that the MCS can be regenerated and reused, which contribute to the cost savings in a large scale (Fig. 6e). Fig. 6f demonstrates the changes of pore-size distribution of the MCS before and after 10 adsorption–desorption cycles. After 10 cycles, the pore-area of the mesopores in the characteristic diameter of ∼50 Å had a certain decrease, compared with the initial state of the MCS, due to that the tiny amounts of Cr(vi) attached to the surface and then lead to the clogging of mesopores. Fig. 7a–e show the process of highly efficient Cr(vi) removal visually (Video S1†) and the solution after Cr(vi) removal could be applied directly for water culture experiment (Fig. 7g–i). Moreover, the PPM-C maintained the stability structure after Cr(vi) removal so that the adsorbent was removed from the wastewater without causing second-pollution (the inset of Fig. 7f).

### Cr(vi) removal percentage at different conditions

**Fig. 8:**
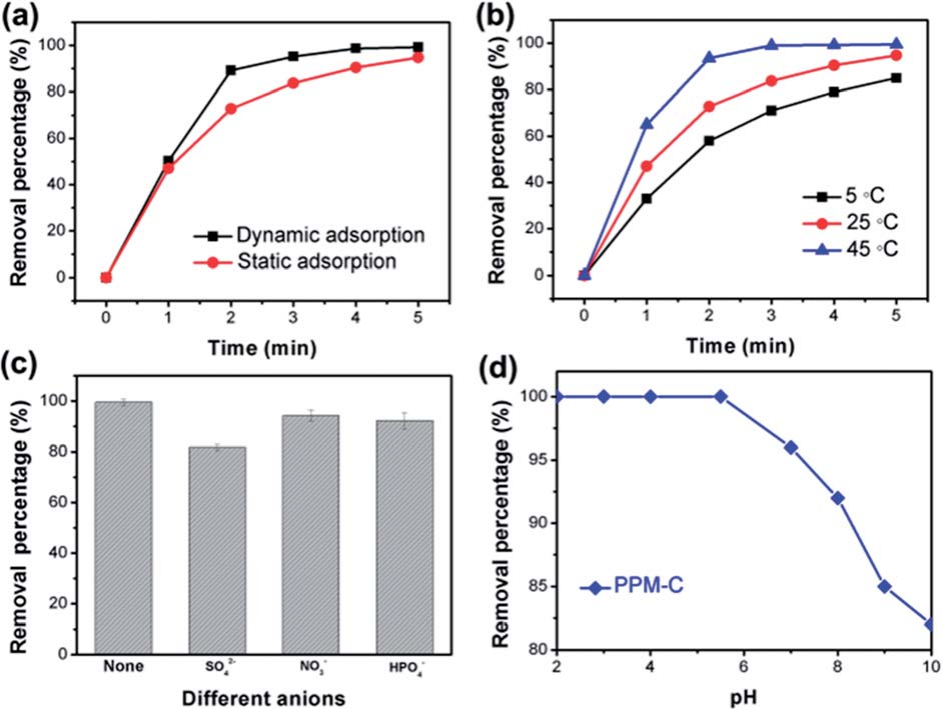
(a) The Cr(vi) RP of PPM-C *vs.* time under dynamic adsorption (100 rpm) and static adsorption at room temperature (25 °C), (b) the Cr(vi) RP of PPM-C *vs.* time at different temperature (under static adsorption), (c) effect of competing anions, and (d) solution pH on the Cr(vi) RP of PPM-C (the concentration of SO_4_^2−^ (Na_2_SO_4_), NO_3_^−^ (NaNO_3_), and H_2_PO_4_^−^ (KH_2_PO_4_) is 0.01 M). (PPM-C for the Cr(vi) removal at different conditions is PP15-M1.5-C).

Fig. 8a displayed the faster Cr(vi) removal of PPM-C under dynamic adsorption compared with the PPM-C under static adsorption, indicating that the mobility of the water contributes to highly efficient Cr(vi) removal. It was found that the temperature is also one of the most important variables affecting the Cr(vi) removal efficiency. As displayed in Fig. 8b, the RP of PPM-C reached to ∼100% at 45 °C, while the RP barely increased to 86.7% at 5 °C. Therefore, the high temperature had positive influence on the Cr(vi) removal process, ascribing to the promoting effect of the higher temperature for efficient ions transfer. Besides, different anions in the wastewater can affect the Cr(vi) removal. The effect of anions in different valences (divalent and monovalent) on Cr(vi) removal was shown in Fig. 8c. The inhibition of the divalent anions (SO_4_^2−^) on the Cr(vi) removal is much more serious than that of the monovalent anions (NO_3_^−^ and H_2_PO_4_^−^), which could be explained by the divalent anions with a higher ion charge density compared with monovalent anions,^49^ making a better affinity of the divalent anions toward adsorbent. To further investigate the influence of solution pH for the adsorption characteristics, the Cr(vi) RP of the PPM-C in solutions of different pH is shown in Fig. 8d. One can observe that the complete Cr(vi) removal was achieved under acidic conditions when the pH was controlled at 2, 3, 4, and 5.5 within 5 min. Increasing the solution pH to 8 and even higher, the Cr(vi) RP sharply decreased to 81.72%. This result may be interpreted that the large amount of OH^−^ ions under alkaline conditions compete with Cr(vi) species on the MCS surface, leading to the decline of Cr(vi) RP.

## Conclusions

In summary, we have manufactured a MCS from one-pot synthesis method of a supramolecular microcrystalline cellulose–polymer system by microwave-assisted heating/annealing treatments. The as-prepared products exhibited high adsorption capacity of 93.96 mg g^−1^ and fast Cr(vi) removal from wastewater only within 5 min. Our studies suggest that the key to obtain a very large removal capacity of Cr(vi) and a fast removal rate is the synergistic effect of the high specific surface area and multilevel structures that consist of 3D interconnected mesopores and a rough interface decorated with ball-like protuberance. More significantly, the carbon sponge are recyclable without causing second-pollution, and thus has great potential applications in environmental and water purification fields.

## Conflicts of interest

There are no conflicts to declare.

## Supplementary Material

RA-008-C8RA00012C-s001

RA-008-C8RA00012C-s002
