# Supplementary material for: A microwave synthesized mesoporous carbon sponge as an efficient adsorbent for Cr(vi) removal
Source: RSC Adv. 2018 Feb 19;8(14):7892–8. doi: 10.1039/c8ra00012c (PMC9078492; doi:10.1039/c8ra00012c)
Supplement: RA-008-C8RA00012C-s001 [file RA-008-C8RA00012C-s001.pdf]

Electronic Supplementary Material (ESI) for RSC Advances.
This journal is © The Royal Society of Chemistry 2018

Supporting Information

Microwave synthetic mesoporous carbon sponge as an efficient adsorbent for Cr(VI) removal

Yan-Jun Liu[a], Shan Liu[a], Zhi-Wen Li[a], Ming-Guo Ma[a,b*], and Bo Wang[a]

[a]*Engineering Research Center of Forestry Biomass Materials and Bioenergy, Beijing Key Laboratory of Lignocellulosic Chemistry, College of Materials Science and Technology, Beijing Forestry University, Beijing 100083, PR China;*

[b]*Key Laboratory of Pulp and Paper Science & Technology of Ministry of Education/Shandong Province, Qilu University of Technology, Jinan 250353, PR China.*

Figure S1. UV-vis absorption of the Cr (VI) solution treated with different MCS (1.0 g L^{-1}) within five minutes.

Figure S2. Mechanisms of electrophilic rearrangement of PVA–OH and PVP–C═O.

Figure S3. EDS images of the composition of MCS (PPN-C).

Figure S4. UV-vis absorption of the Cr (VI) solution treated with different biochar sponges.

Table S1. Composition of carbon sponges.

Table S2. Comparison of adsorption capacity and rate constant with other materials.

*Corresponding author. Tel.: +86-10-62337250, Fax.: +86-10-62336903.
E-mail address: mg_ma@bjfu.edu.cn (M.-G. Ma)

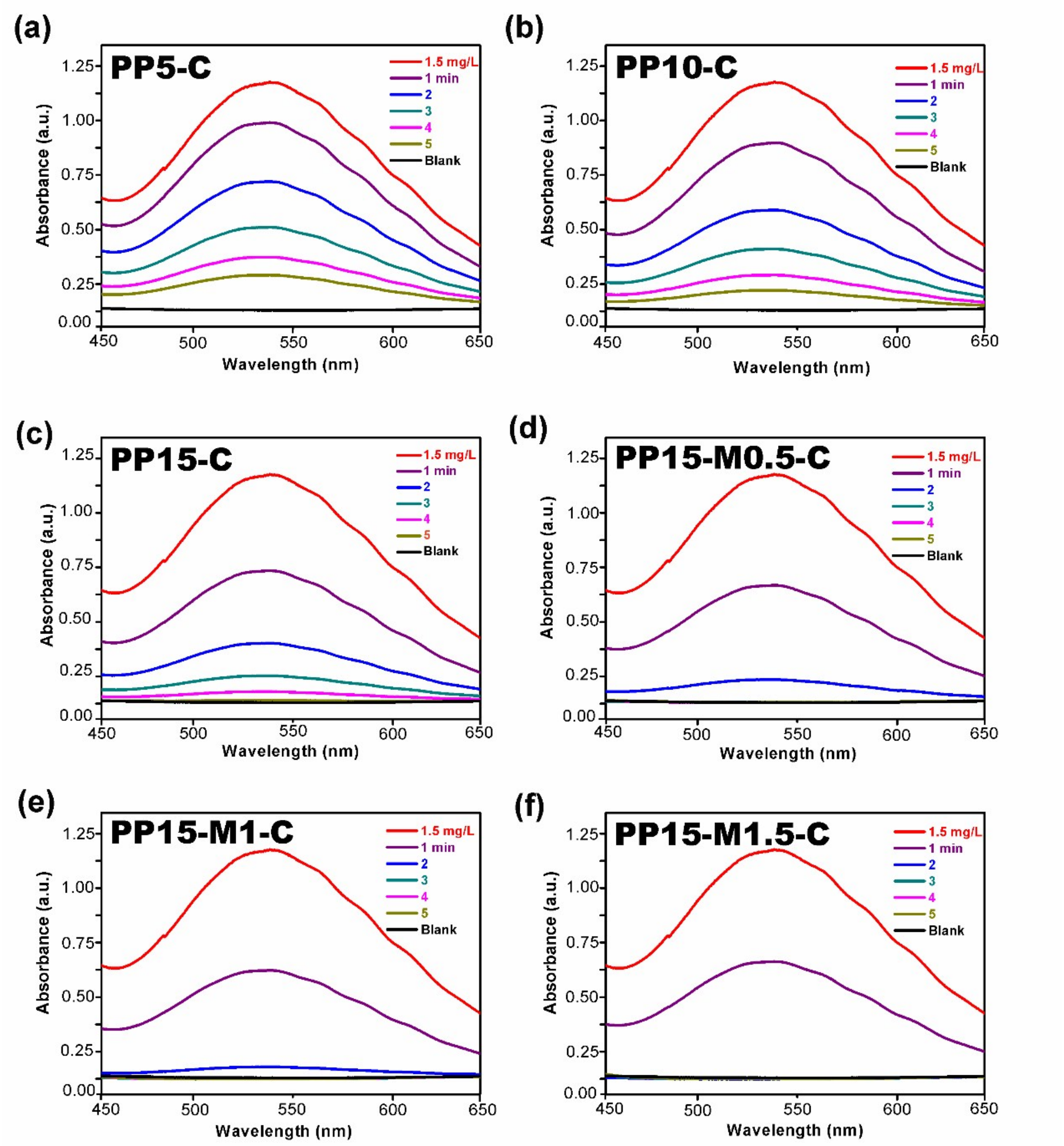

Figure S1. UV-vis absorption of the Cr (VI) solution treated with MCS (1 g L^{-1}) within five minutes.

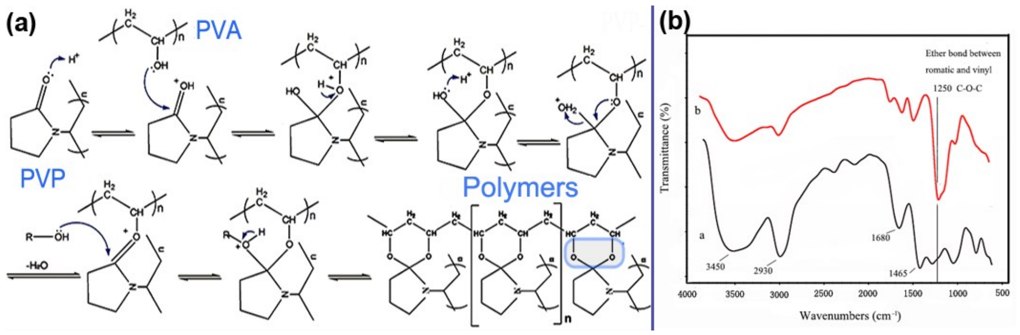

Figure S2. (a) Electrophilic rearrangement of PVA–OH and PVP–C═O to generate homogemeous

network polymers; (b) FT-IR spectra of the polymers (a) before and (b) after self-assembly.

In this work, the alcoholic hydroxyl side-groups of the PVA chain and ketone groups of the PVP chain induced the self-assembly under acid catalysis, obtaining a 1,3-dioxane polymer (Fig. S2a). As shown in the FT-IR spectra (Fig. S2b), the absorptions of the –C═O (1680 cm $^{-1}$) stretch at 1680 cm $^{-1}$ and the OH (3450 cm $^{-1}$) stretch located at 3450 cm $^{-1}$ decreased after the self-assembly reaction. A new absorption peak of the C–O–C stretch appeared at 1250 cm $^{-1}$, indicating that the functional groups were consumed along with an appearance of new functional group during the reaction.[1,2]

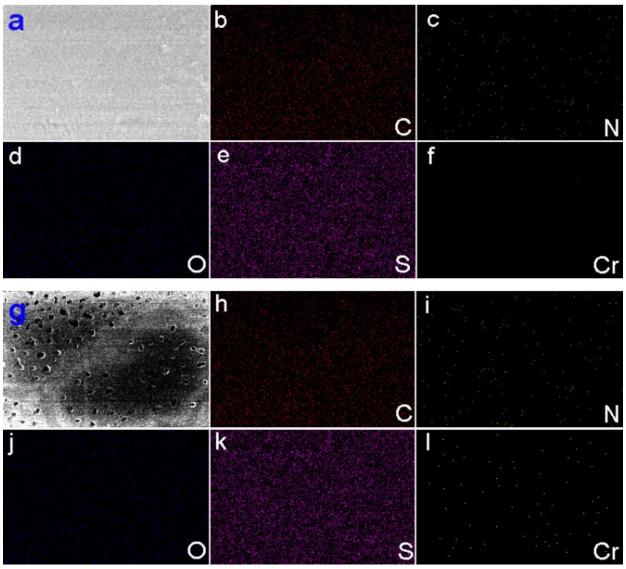

Figure S3. EDS images of the composition of MCS (PPN-C) (a)-(f) before and (g)-(l) after Cr(VI) removal (b/h C map, c/i N map, d/j O map, e/k S map, f/l Cr map).

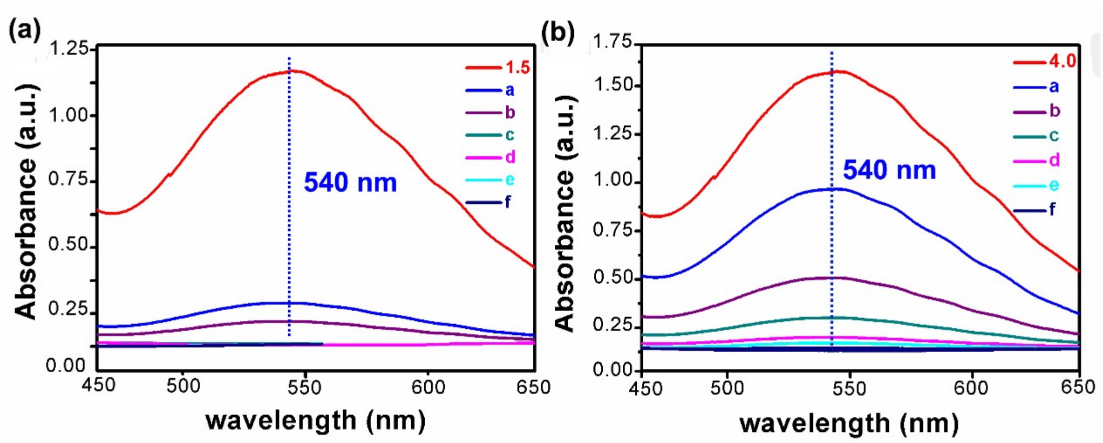

Figure S4. (a) and (b) UV-vis absorption of the Cr (VI) solution treated with different biochar sponges (Adsorbent: 1.0 mg L^{-1}, Cr(VI): 1.5 mg L^{-1} and 4.0 mg L^{-1}, treating time: 5 min). (a, b, c, d, e, and f represents PP5-C, PP10-C, PP15-C, PP15-M0.5-C, PP15-M1-C, and PP15-M1.5-C, respectively).

Table S1. Composition of carbon sponges.

Code	[a]ω_{PVA} (%)	[a]ω_{PVP} (%)	V_{PVA}:V_{PVP}	[b]ω_{MCC} (%)	[c]$\omega_{sulfuric\ acid}$ (%)
PP5-C	8	5	7:3	—	1.5
PP10-C	8	10	7:3	—	1.5
PP15-C	8	15	7:3	—	1.5
PP15-M0.5-C	8	15	7:3	0.5	1.5
PP15-M1-C	8	15	7:3	1	1.5
PP15-M1.5-C	8	15	7:3	1.5	1.5

[a]The mass fraction (ω) was calculated by the following equation, $\omega = [m_{solute}/(m_{solute+mdeionized\ water}]\times 100\%$. [b]The mass fraction (ω) was calculated by the following equation, $\omega = [m_{MCC}/(m_{MCC}+m_{(mixture\ of\ PVA\ and\ PVP)})]\times 100\%$, [c]The mass fraction (ω) was calculated by the following equation, $\omega = [m_{10\%\ sulfuric\ acid}/(m_{10\%\ sulfuric\ acid}+m_{(mixture\ of\ PVA\ and\ PVP)})]\times 100\%$.

Table S2. Comparison of adsorption capacity and rate constant with other materials.

Materials	Adsorption capacity (mg g^{-1})	Adsorption rate (mg g^{-1} min $^{-1}$)	Ref.
MCS (in this work)	93.96	18.79	
Core@double-shell nanoparticles	0.75	0.15	3
Magnetic carbon nanocomposite fabrics	3.74	0.38	4
Aluminum–magnesium mixed	105.3–112.0 [a]	N/A	5
PAMAM-g-CNFs	377.36[b]	~ 0.62	6
Activated carbon	112.36[c]	<0. 93	7

[a]The large adsorption capacity of aluminum–magnesium mixed hydroxide is due to the ion exchange mechanism rather than adsorption which results in the very low adsorption rate. [b] In the process of Cr(VI) removal, the part of Cr(VI) ions been reduced to Cr(III), which meant that PAMAM-g-CNFs for Cr(VI) removal is chemisorption and the adsorption kinetics doesn't follows the pseudo-second-order model. [c]Activated carbon exhibits a very high specific adsorption capacity due to their extremely low densities. Nanocomposites with metal oxides have significantly higher densities. Therefore, the specific adsorption capacity based onunit mass is much lower.

REFERENCES

(1) Gao, L.; Han, J. G.; Lei, X. G. *Org. Lett.* **2016**, *18*, 360-363.

(2) Kumar, R.; Kumar, D.; Chakraborti, A. K. *Synthesis* **2007**, *2*, 299-303.

(3) Zhu, J. H.; Wei, S. Y.; Gu, H. B.; Rapole, S. B.; Wang, Q.; Luo, Z. P.; Haldolaarachchige, N.; Young, D. P.; Guo, Z. H. *Environ. Sci. Technol.* **2012**, *46*, 977-985.

(4) Zhu, J. H.; Gu, H. B.; Guo, J.; Chen, M. J.; Wei, H. G.; Luo, Z. P. *J. Mater. Chem. A* **2014**, *2*, 2256-2265.

(5) Li, Y.; Gao, B.; Wu, T.; Sun, D.; Li, X.; Wang, B.; Lu, F. *Water Res.* **2009**, *43*, 3067-3075.

(6) Zhao, J.; Zhang, X.; He, X.; Xiao, M.; Zhang, W.; Lu, C. *J. Mater. Chem. A* **2015**, *3*, 14703-14711.

(7) El-Sikaily, A.; Nemr, A. E.; Khaled, A.; Abdelwehab, O. *J. Hazard. Mater.* **2007**, *148*, 216-228.
